# Adrenal Insufficiency following Stereotactic Ablative Radiotherapy (SAbR) of Adrenal Gland Metastases

**DOI:** 10.3390/cancers16183140

**Published:** 2024-09-12

**Authors:** Oksana Hamidi, Mihailo Miljanic, Gayane Tumyan, Alana Christie, Sasan Mirfakhraee, Sadia Ali, Michael Dohopolski, Sujana Gottumukkala, James Brugarolas, Robert Timmerman, Raquibul Hannan

**Affiliations:** 1 Division of Endocrinology and Metabolism, University of Texas Southwestern Medical Center, Dallas, TX 75390, USA; oksana.hamidi@utsouthwestern.edu (O.H.); gayane.tumyan@utsouthwestern.edu (G.T.); sasan.mirfakhraee@utsouthwestern.edu (S.M.); sadia.ali@utsouthwestern.edu (S.A.); 2 Department of Radiation Oncology, University of Texas Southwestern Medical Center, Dallas, TX 75390, USA; mihailo.miljanic@utsouthwestern.edu (M.M.); robert.timmerman@utsouthwestern.edu (R.T.); 3 Kidney Cancer Program, Simmons Comprehensive Cancer Center, University of Texas Southwestern Medical Center, Dallas, TX 75390, USA; alana.christie@utsouthwestern.edu (A.C.); michael.dohopolski@utsouthwestern.edu (M.D.); sujana.gottumukkala@utsouthwestern.edu (S.G.); james.brugarolas@utsouthwestern.edu (J.B.); 4 Department of Internal Medicine, Hematology-Oncology Division, University of Texas Southwestern Medical Center, Dallas, TX 75390, USA

**Keywords:** adrenal metastasis, adrenal insufficiency, radiotherapy, SBRT, SAbR, stereotactic body radiation therapy, adrenalectomy, renal cell carcinoma, adrenal lesion, adverse event

## Abstract

**Simple Summary:**

Stereotactic ablative radiation (SAbR) is a focused, high-dose radiation technique used to treat cancers that have spread to the adrenal glands. One of the feared consequences of this therapy is causing adrenal insufficiency in patients, which is a lack of adrenal function; however, the rates and severity at which this occurs in patients are still poorly studied. We studied patients with 66 treated adrenal glands using SAbR, which demonstrated that this technique was effective at controlling cancer sites with a control rate of 75% at 1 year. The risk of patients developing adrenal insufficiency in the entire cohort was significant at 14%, with a median time of 4.3 months. There was a higher risk in patients who had both adrenal glands treated with SAbR, or who had received a prior surgical removal of their other adrenal gland prior to SAbR therapy, with 44% of these patients developing adrenal insufficiency.

**Abstract:**

Background: Adrenal metastases are often treated with stereotactic ablative radiation (SAbR). We aimed to assess the incidence, timing, and factors associated with the development of primary adrenal insufficiency (PAI) following SAbR. Methods: A retrospective cohort study comprised 66 consecutive patients (73% men, median age 61 years) who underwent SAbR for adrenal metastasis. Results: The series encompassed metastases from renal cell carcinoma (41%), lung tumors (38%), colorectal adenocarcinoma (9%), melanoma (5%), and others (7%). Median follow-up was 17 months from SAbR. Nine (14%) patients developed PAI at a median of 4.3 months (range, 0.7–20.2). The incidence of PAI was 44% in patients with prior adrenalectomy receiving unilateral SAbR, 44% with bilateral SAbR, 2% with unaffected contralateral gland, and 0% with bilateral metastases treated with unilateral SAbR. PAI was associated with prior adrenalectomy (odds ratio [OR] 32) and bilateral SAbR (OR 8.2), but not age, sex, metastasis size, or biological effective dose. Post-SAbR 6-month and 1-year local control rates were 82% and 75%, respectively. Conclusions: Patients undergoing SAbR for adrenal metastasis are at high risk of developing PAI. PAI is associated with bilateral SAbR and contralateral adrenalectomy. PAI is unlikely with a remaining unaffected adrenal gland or in the setting of bilateral adrenal metastases with unilateral SAbR.

## 1. Introduction

Adrenal metastasis is the second most common cause of neoplasms of the adrenal cortex, accounting for more than 90% of secondary tumors [1]. Due to their abundant vascular supply, the adrenal glands provide a favorable environment for metastatic infiltration. Metastases account for 30% to 70% of adrenal masses in patients with prior diagnosis of cancer or active extra-adrenal malignancy, particularly in the setting of bilateral adrenal involvement [2]. The prevalence of bilateral adrenal metastases ranges between 4% and 20% [3,4,5]. Certain malignancies, including lung, breast, gastrointestinal, renal, and malignant melanoma, have a propensity to metastasize to the adrenal glands [6,7].

Primary adrenal insufficiency (PAI) typically develops when more than 90% of the adrenocortical tissue is compromised [6,8]. PAI in a setting of metastatic malignancy can result from tumor infiltration, systemic therapies (immunotherapy), surgery, and minimally or non-invasive procedures. Up to 12.4% of patients with bilateral adrenal metastases are reported to develop PAI, with up to 20% of patients with a large tumor size >4 cm [9]. Furthermore, patients affected by cancer are also at risk for secondary adrenal insufficiency related to the use of immunotherapy that can cause hypophysitis or high-dose systemic glucocorticoids that can cause the suppression of the hypothalamic–pituitary–adrenal axis.

Treatment modalities for adrenal metastasis include systemic therapy and local control measures, including adrenalectomy, radiofrequency ablation (RFA), microwave ablation, cryoablation, and stereotactic ablative radiation (SAbR) [10,11,12,13,14]. Local ablative measures for adrenal metastasis are frequently utilized in the treatment paradigm for patients with oligometastatic or oligoprogressive disease [15,16,17,18,19]. Randomized data from prospective studies demonstrated that the addition of local ablative measures to metastasis improves not only progression-free survival but also overall survival in selected patients [20,21]. SAbR for adrenal metastasis has been shown to be well tolerated and exhibits low toxicity rates, with grade 2 toxicity ranging between 0% and 15% [22]. SAbR to treat oligometastatic renal cell carcinoma has been shown to provide local control rates of greater than 85% [16,18,23].

Although generally well tolerated, SAbR for adrenal metastases can pose further risk for developing PAI in patients with an already compromised adrenal anatomy in the setting of adrenal metastasis and/or prior surgical intervention. If unrecognized, PAI can be life-threatening. Therefore, it is critical to understand the incidence and predictors of PAI in patients with adrenal metastasis undergoing local treatment, and how it could impact patient management and outcomes.

PAI due to radiation treatment has been previously reported in case reports or case series [12,13,24,25]; however, data regarding SAbR-specific effects on adrenal function remain limited. Therefore, the primary objective of our study was to assess the incidence of PAI in patients undergoing SAbR for the treatment of adrenal metastases. Additionally, we aimed to identify risk factors and the timing of development of PAI following SAbR treatment.

## 2. Subjects and Methods

### 2.1. Subjects

Utilizing an IRB-approved registry protocol, we performed a retrospective cohort study of consecutive patients who underwent SAbR for treatment of adrenal metastasis at University of Texas Southwestern Medical Center (UTSW) in Dallas, TX, USA between 1 January 2007 and 31 March 2022. The UTSW Radiation Oncology retrospective registry captured all patients treated with SAbR for adrenal metastasis (n = 66) during the study interval. This allowed the identification of all consecutive patients in this clinical context. All electronic medical records were reviewed for data verification and extraction. Patients with pre-SAbR adrenal insufficiency or chronic use of systemic glucocorticoids were excluded from the analysis. All included patients did not have documented evidence of adrenal insufficiency prior to the procedure.

Adrenal metastasis diagnosis was characterized using computed tomography (CT), magnetic resonance imaging (MRI), or positron emission tomography combined with CT (PET-CT). An adrenal gland biopsy for the histopathologic confirmation of the diagnosis of malignancy was performed when clinically indicated.

All patients underwent SAbR, utilizing standard procedures as reported in detail previously [26]. Briefly, planning CT imaging was acquired during the patient-simulation visit, at which time patients were scanned in supine position and immobilized with body frame and vacuum-loc bag setup, with motion assessment and management as indicated. Treatment planning, dose constraints, movement compensation, and treatment delivery were executed as described previously [24]. Local control and progression were defined using follow-up imaging with CT or MRI utilizing Response Evaluation Criteria in Solid Tumors (RECIST) 1.1 criteria. Briefly, RECIST 1.1 criteria define a progressive disease as a ≥20% increase in the sum of longest diameters with an absolute increase of ≥5 mm [27].

The SAbR intent was defined using the following criteria: consolidative—radiation was given in conjunction with or concurrent with systemic therapy in the setting of oligoprogression; curative—when there was only one site of metastasis and radiation given with the intent to cure the patient; definitive—when there was oligometastasis (1–5) and radiation was given with the intent to provide maximum local control of the treated site; and palliative—radiation was given for the purpose of palliation of sign or symptom.

PAI was defined in accordance with the Endocrine Society guidelines [28]. PAI was diagnosed when early morning serum cortisol concentration was <5 μg/dL in combination with a plasma adrenocorticotropic hormone (ACTH) quantity greater than 2-fold the upper limit of the reference range [28,29]. The cosyntropin stimulation test (ACTH 1–24) was carried out in patients with indeterminate serum cortisol values if the confirmation of PAI was needed (i.e., if the early morning serum cortisol concentration was >5 μg/dL but <18 μg/dL), or per discretion of the treatment provider. A peak serum cortisol concentration lower than 18 μg/dL at 30 or 60 min, or alternatively a new serum cortisol cutoff of 14.5 μg/dL at 30 min depending on the assay used, were consistent with the diagnosis of PAI [28,29]. Mineralocorticoid insufficiency was diagnosed when serum aldosterone was low and renin level was elevated. Serum cortisol levels were measured using the Abbott Alinity system (Abbott Laboratories, Abbott Park, IL, USA). ACTH measurement was performed on the Roche cobas quantitative electrochemiluminescent immunoassay system (Roche Cobas, Indianapolis, IN, USA). Aldosterone and plasma renin activity levels were measured using an immunochemiluminescent assay by a reference laboratory (Mayo Clinic Laboratories, Rochester, MN, USA).

Upon establishing PAI diagnosis, treatment with glucocorticoid replacement therapy typically with 15–25 mg/day of hydrocortisone (in two divided doses) was initiated, unless the patient presented with adrenal crisis, in which case a hydrocortisone bolus of 100 mg was delivered intravenously, followed by 200 mg over 24 h. Fludrocortisone treatment was initiated when mineralocorticoid deficiency was present [30].

The Common Terminology Criteria for Adverse Events (CTCAE) version 5.0 was utilized for AI grading: Grade 1, asymptomatic, clinical, or diagnostic observations only, intervention not indicated; Grade 2, moderate symptoms, medical intervention indicated; Grade 3, severe symptoms, hospitalization indicated; Grade 4, life-threatening consequences, urgent intervention indicated; Grade 5, death. With one exception, none of the patients were on exogenous glucocorticoids prior to the diagnosis of PAI to ensure the accurate assessment of adrenal function. One patient treated with chronic oral dexamethasone was excluded from the PAI analysis.

### 2.2. Data Analysis

Descriptive statistics were used to provide a summary of the data. Categorical data were reported as absolute and relative frequencies (percentages). Continuous data were presented as median (interquartile range or minimum to maximum range, as specified). To compare medians between two independent groups, we used the nonparametric Wilcoxon rank sum test. Logistic regression models were used to estimate the association of factors in the development of PAI. All tests were two-sided, and *p* values less than 0.05 were considered statistically significant. Survival probability was assessed using the Kaplan–Meier estimator, starting from the first day of SAbR. All statistical analyses were conducted using SAS version 9.4 (SAS Institute Inc., Cary, NC, USA).

## 3. Results

### 3.1. Patient Characteristics

In this study, we report the demographics, the clinical characteristics including stage and primary malignancy, treatment, and the outcomes of 66 patients (73% men; median age, 61 years) with adrenal metastasis treated with SAbR (Table 1). The median follow-up was 50.2 months from the initial cancer diagnosis (range, 5.8–186.4) and 17 months from SAbR (range, 2.7–89.8). Primary carcinomas included kidney (41%), lung (38%), and colorectal (9%).

Overall, 41 patients (62%) had unilateral adrenal gland metastasis, while 11 patients (16.7%) had synchronous adrenal metastatic involvement (the simultaneous discovery of bilateral adrenal metastases), and 14 (21.2%) had metachronous adrenal involvement. The median time to development of adrenal metastasis was 14 months, and the median time to the contralateral adrenal metastasis was 6 months in patients with metachronous adrenal metastases. Eleven (16.7%) patients underwent unilateral adrenalectomy for adrenal metastasis (Table 2).

### 3.2. SAbR for Adrenal Metastasis

At the time of SAbR, 41 (62%) patients had an unaffected contralateral adrenal gland, 16 (24%) had bilateral adrenal metastases, and 9 (14%) underwent contralateral adrenalectomy prior to SAbR (Table 2). Adrenal SAbR was delivered to patients at a median time of 30 months (16–53) from the initial cancer diagnosis to the first SAbR treatment, with 9 patients (13.6%) receiving bilateral adrenal SAbR, 25 patients (37.9%) receiving SAbR to the left adrenal gland, and 32 patients (48.5%) receiving SAbR to the right adrenal gland (Table 2).

### 3.3. Factors Associated with Post-SAbR PAI

**Figure 1:**
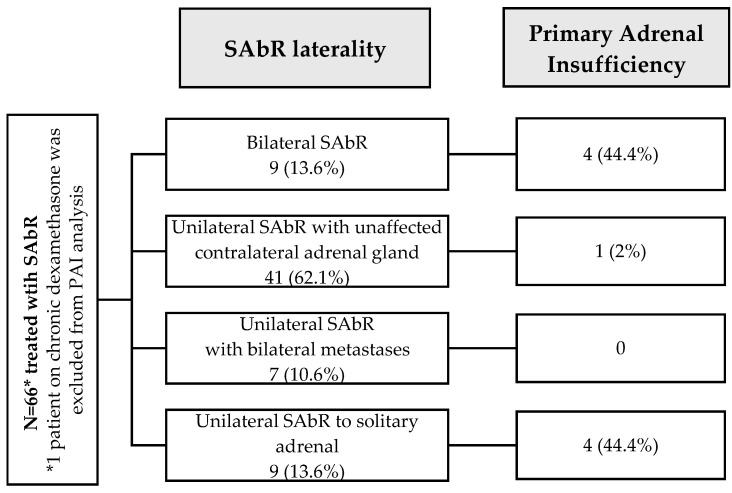
Development of primary adrenal insufficiency with patients treated with SAbR for adrenal metastasis.

A total of 9 (13.8%) patients in our cohort were diagnosed with post-SAbR PAI; 44% (4/9) of patients were diagnosed with bilateral SAbR, 44% (4/9) with prior contralateral adrenalectomy, only 2% (1/41) had an unaffected contralateral adrenal gland, and 0% (0/7) had unilateral SAbR in the setting of bilateral adrenal metastases (Table 3 and Table 4, Figure 1). Of these, three patients presented with adrenal crisis (Pt 4 with CTCAE 3 and Pts 1, 6 with CTCAE 4). Four patients developed mineralocorticoid deficiency and were treated with fludrocortisone. None of the patients with bilateral adrenal metastases who were treated with unilateral SAbR developed PAI at the conclusion of the study.

**Figure 2:**
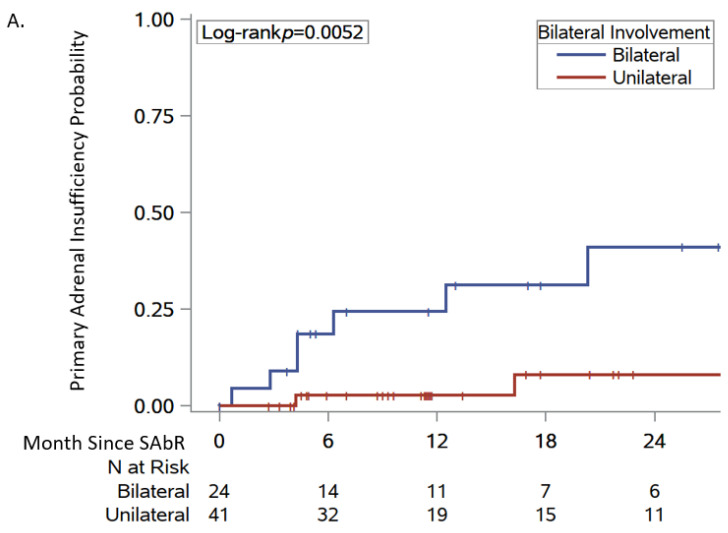

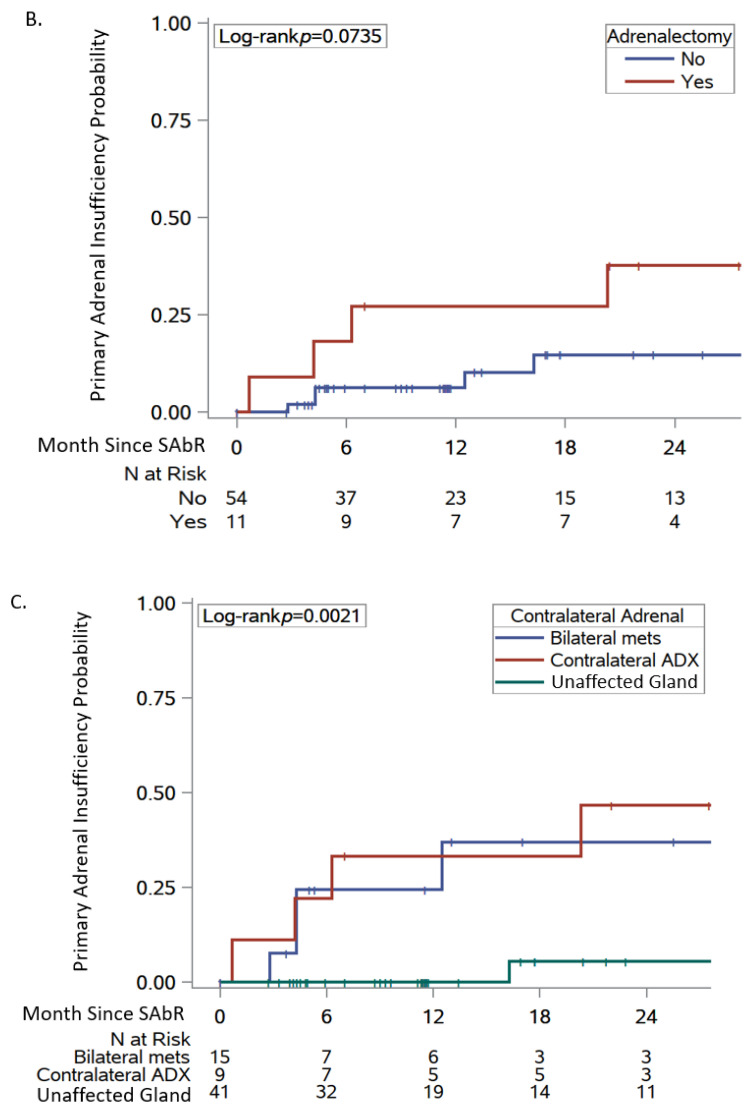

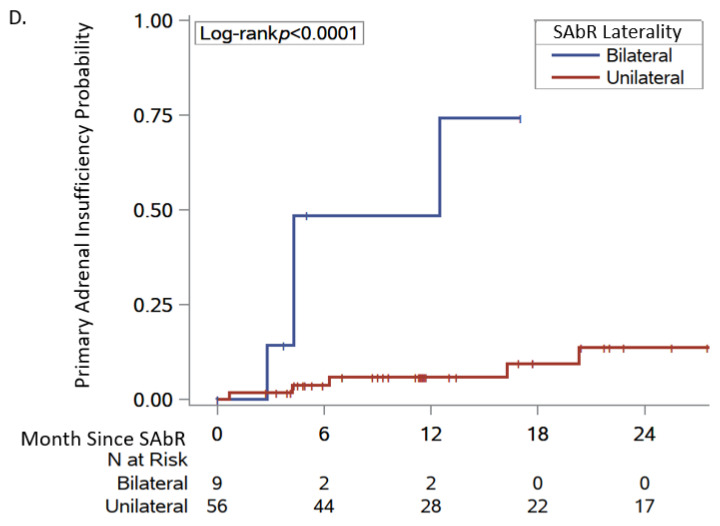
Probability of post-SAbR primary adrenal insufficiency in patients with unilateral and bilateral metastases (Panel (**A**)), adrenalectomy (Panel (**B**)), status of contralateral adrenal gland at the time of SAbR (Panel (**C**)), and laterality of SAbR treatment (Panel (**D**)).

**Figure 3:**
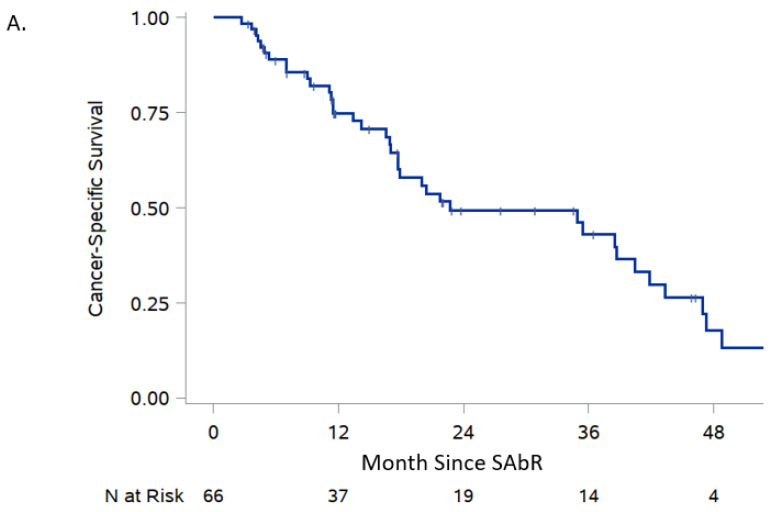

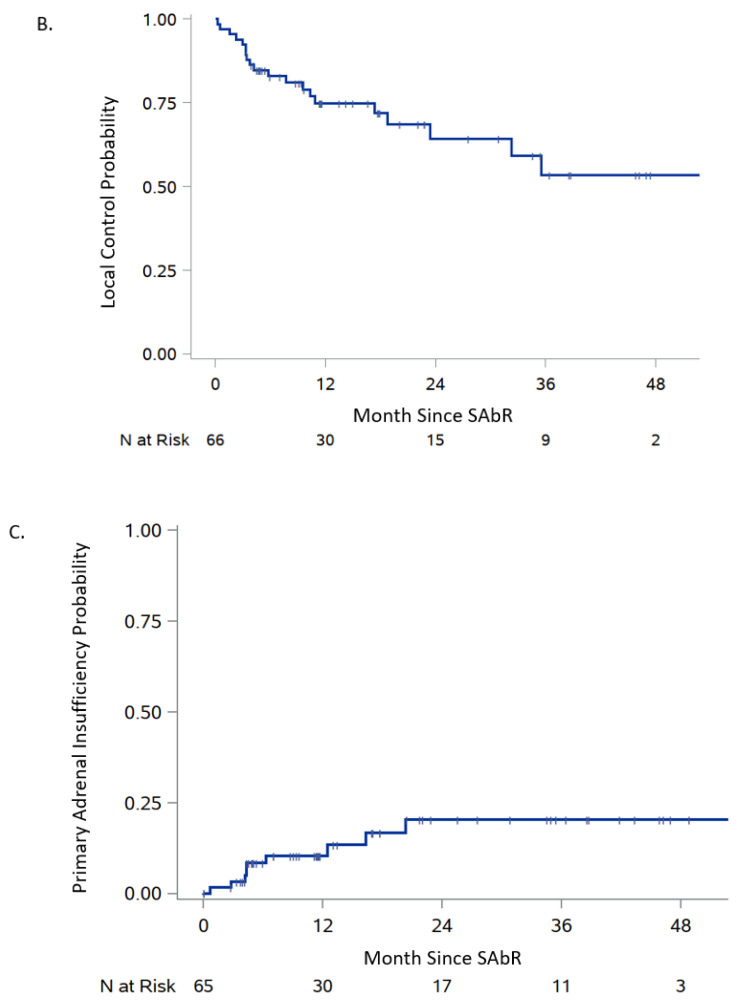

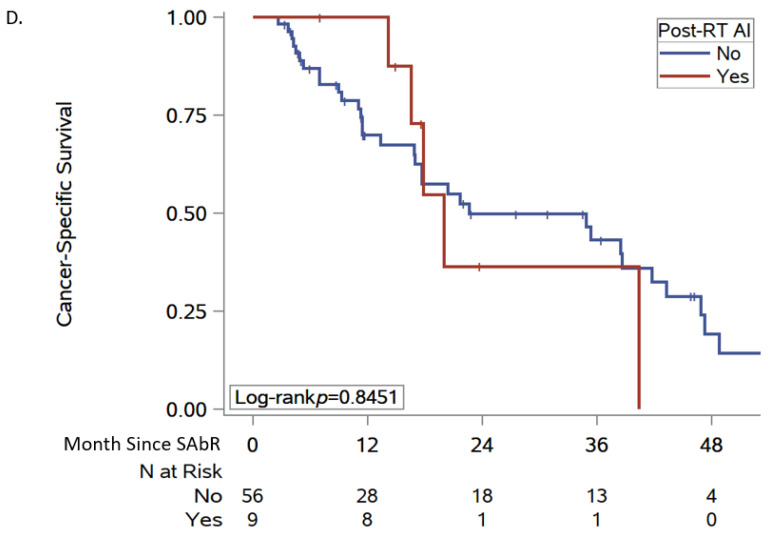
Cancer-specific survival (Panel (**A**)), local metastasis control (Panel (**B**)), overall development of primary adrenal insufficiency (Panel (**C**)), and cancer specific survival with and without adrenal insufficiency (Panel (**D**)) following SAbR.

The median time to the development of PAI was 4.3 months (range, 0.7–20.2) (Figure 2 and Figure 3). The risk factors associated with PAI were prior contralateral adrenalectomy (odds ratio [OR] 32, 95% confidence interval [CI] 2.96–346) and SAbR treatment to bilateral metastases (OR 8.16, 95% CI 1.64–40.6) (Figure 2C). In contrast, age, sex, tumor size, and pre-SAbR treatments were not associated with the development of PAI (Table 3 and Table 4). Six-month and 1-year local control rates were 82.9% and 74.8%, respectively (Table 5, Figure 3B). Following SAbR, we observed 6-month and 1-year overall survival rates of 89% and 74.8%, respectively (Figure 3A).

Of nine patients with PAI, seven received systemic therapy during their treatment course (78%), five patients received systemic chemotherapy or targeted therapy (56%), four patients (44%) received immunotherapy, and one patient (11%) was on chronic systemic glucocorticoid therapy for bullous pemphigoid. Two patients (22%) developed grade 4 toxicity due to PAI. In both patients, PAI developed later than for most other patients (12.5 and 20.2 months after SAbR). Both patients with grade 4 toxicity were treated with a course of 40 Gy in 5 Fx, and required treatment for PAI with hydrocortisone and fludrocortisone. One patient died 5 months following PAI diagnosis due to cancer progression. One additional patient developed grade 3 PAI toxicity and received hydrocortisone replacement, with six (67%) patients with PAI exhibiting grade 1–2 toxicity. Two of these patients were admitted to hospice care following adrenal SAbR, both due to progression of disease.

## 4. Discussion

Our findings showed that patients treated with SAbR for adrenal metastasis are at risk for developing PAI (13.8% in our cohort). Patients with prior contralateral adrenalectomy and SAbR to bilateral adrenal metastases were at the highest risk for PAI (44% in both groups), compared to those with bilateral metastasis treated with unilateral SAbR (0%). Notably, bilateral adrenal SAbR was associated with a 44% risk of PAI, which is contrary to the 100% expected from bilateral adrenalectomy. However, it remains to be determined whether PAI may develop with longer follow-up (or prolonged life expectancy). Nonetheless, compared to surgery, where PAI is immediate, SAbR can preserve adrenal function for a longer period. This suggests that maximal sparing of the radiated adrenal gland likely keeps it functional through avoiding the development of PAI. There is also likely a radiation dose threshold, which is yet unknown, below which it may continue to function.

Both adrenalectomy and radiation therapy to adrenal glands have been linked to the development of PAI [11,12,13,24,25,31]. This is in contrast to what we report in our series where the risk of developing PAI is 0–2% when one adrenal metastasis is treated with radiation and the other one is spared an intervention. Admittedly, the development of PAI is multifactorial and patients with diabetes, hypertension, obesity, and larger tumors were more likely to develop PAI in previous series [11].

Factors associated with the development of PAI in our study included prior adrenalectomy and bilateral SAbR. Compatible findings were noted in a prior study, demonstrating that bilateral adrenal radiotherapy or unilateral adrenal radiotherapy in the setting of the solitary adrenal gland increased the risk of developing PAI up to 80% [25]. Similarly, the study showed an increased risk of PAI with bilateral as opposed to unilateral focal adrenal treatment and in those that receive bilateral adrenal radiation [25]. A strong association between prior adrenalectomy and the development of PAI following SAbR (as demonstrated in our study as well as prior study [25]) is likely explained by a compromised adrenal reserve. Since PAI typically develops when more than 90% of the adrenal tissue is compromised [6,8], adrenalectomy potentially heightens the risk for PAI by effectively reducing residual adrenal tissue. Another possible explanation for this association is compromised vascular supply. A similar phenomenon has been thoroughly described in women who have an increased risk of ovarian failure after undergoing hysterectomy [32]. Although the exact mechanisms are not known, one potential explanation is a compromise on ovarian blood supply in the process of hysterectomy. In addition, the increased risk for PAI patients with bilateral SAbR is likely multifactorial, owing to the effective reduction in the adrenal cortex by metastatic disease and the further destruction of residual adrenal tissue by radiation-mediated cellular and DNA damage [33].

The timing of PAI diagnosis in our cohort showed significant variation, ranging from 3 weeks to 20 months (median 4.3 months) following SAbR, indicating the importance of the close monitoring of adrenal function longitudinally. In a recent study, 14.3% developed PAI with a median time to diagnosis from radiation of 6.1 months [25], concordant with our findings. Thus, it is imperative that patients are counseled on signs and symptoms of impending PAI and undergo periodic biochemical monitoring with 8 AM ACTH and serum cortisol measurements, even in the absence of PAI symptoms. Additional laboratory testing should include electrolyte panel, aldosterone, and plasma renin activity if mineralocorticoid insufficiency is suspected (e.g., dizziness, orthostatic hypotension, salt craving). The counseling and biochemical assessments should ideally be implemented prior to SAbR (particularly in high-risk patients), and then at least every 3–6 months post SAbR, especially during the first 12–24 months. PAI can be overlooked in patients with metastatic malignancy, as many symptoms, including fatigue, weakness, weight loss, anorexia, nausea, or vomiting are frequently present in this patient population due to a combination of primary illness and toxicity from concomitant therapies. Furthermore, impending or untreated PAI can hinder patients’ ability to tolerate necessary treatments, including systemic therapy with chemotherapy and/or immunotherapy. Thus, a high degree of suspicion and systematic assessments of adrenal function need to be maintained in patients with advanced cancers involving the adrenal glands. Early detection and treatment of PAI are imperative to maximize patient outcomes.

In our study, approximately 17% of patients had synchronous adrenal metastatic involvement of the contralateral gland, and 21% exhibited metachronous adrenal metastatic involvement, while the majority of patients had only unilateral adrenal involvement. While the median time to development of a contralateral metastases in this cohort was 6 months, there was a wide range in the timing of contralateral metastasis development, ranging from 0 months to 23 months. Previous studies assessing interventions including adrenalectomy in patients with metachronous vs. synchronous adrenal metastases have demonstrated significantly worse survival in this latter cohort, with contributing factors including the potential of a more aggressive disease or the development of PAI [26]. There was no difference in the risk of PAI development between those with synchronous versus metachronous adrenal involvement, yet the hazard ratio in those with synchronous adrenal metastases was higher, suggesting that the metastases themselves may be interfering with or destroying adrenal function, leading to an increased risk of PAI.

Our overall cohort of patients who received SAbR for adrenal gland metastases demonstrated a PAI-free survival of 80.5% (63.2–90.2) at 6 months, while 6-month PAI-free survival was significantly worse in patients with bilateral adrenal metastasis (61.5%) or in patients with prior contralateral adrenalectomy (64.8%). Conversely, none of the patients with an intact and untreated contralateral adrenal gland developed PAI at 6 months, which is consistent with low historical rates of PAI in these cohorts [8,25].

At 1 year following SAbR, local control was 75.0% (60.8–84.7), and a PAI-free survival was 76.8% (58.6–87.8) in our study. While these are reassuring results compared to the historical data, others have reported less favorable outcomes. A study evaluating survival among 19 patients with adrenal metastasis from lung cancer undergoing SAbR showed the overall survival rates at 1-, 2-, and 5- years to be 56%, 33%, and 22%, respectively [27]. A study evaluating 28 patients with oligometastatic or oligoprogressive malignancy undergoing SAbR for adrenal metastasis showed a 1-year progression-free survival of 26.3% and 1- and 2- year overall survival of 46.6% and 32.0%, respectively [15]. These differences could be due to the inclusion of adrenal metastasis from different primary tumors which can have vastly different rates of progression.

All patients in our cohort were recruited from a single academic institution, which allowed for the uniformity of laboratory assays, reference values, imaging interpretation, and treatment protocol consistency. Yet, this is a limitation, since it may compromise the generalizability of the findings. Our retrospective analysis has several additional limitations. Although we included all consecutive patients treated with SAbR in our center, not all patients were systematically evaluated for adrenal insufficiency, which could have resulted in an underestimation of PAI incidence. The total number of patients in our study is small and the study cohort is heterogenous. Some patients in our study received other pre-SAbR treatments, including immunotherapy and chemotherapy, which can be potential confounders for the development of PAI. For patients with bilateral adrenal metastases who received adrenal radiation, it is difficult to attribute causality, as affected patients may be predisposed to PAI at baseline due to the destruction of the adrenal cortex by metastatic process. The lack of association with age, sex, metastasis size, or dose could potentially be explained by a type II error due to an underpowered sample size. Although not found to be statistically significant in our study, these are important clinical variables that could potentially assist with identifying at-risk patients. Although we did not assess other local treatments in our study, our findings can potentially be applicable to other local treatments for adrenal metastasis, such as thermal ablative techniques or other types of primary malignancies. Lastly, we did not include a control group of patients who underwent adrenal metastasis treatment other than SAbR, which further limits the impact of our findings.

## 5. Conclusions

In conclusion, our study showed that patients with a surgically absent contralateral adrenal gland, bilateral metastases, and bilateral SAbR are at high risk for developing PAI. The risk of development of PAI in this setting is not a certainty, but ranges from 27 to 44% and may be higher with longer follow-up. Therefore, this patient population benefits from the early assessment of adrenal function (within 2–4 weeks) throughout the radiation treatment and warrants close monitoring by an endocrinologist following treatment delivery. Patients with intact contralateral adrenal glands appear to have more favorable outcomes with a lower risk of PAI. This study suggests that a multi-disciplinary approach to the management of patients with adrenal metastasis is vital to the detection and treatment of PAI following SAbR. Future research should focus on prospective studies or trials that could confirm the findings and address unanswered questions, such as other risk factors for the development of PAI, optimal monitoring strategies for PAI post-SAbR, and the long-term outcomes of patients who develop PAI.

## Figures and Tables

**Table 1:** Patient characteristics.

	N (%) (n = 66)
Median age at cancer diagnosis (IQR)	61 (55–66)
Sex	
Male	48 (73%)
Female	18 (27%)
Primary tumor site	
Kidney	27 (41%)
Lung	25 (38%)
Colorectal	6 (9%)
Melanoma	3 (5%)
Bladder	2 (3%)
Angiosarcoma	1 (1.5%)
Hepatocellular	1 (1.5%)
Thyroid	1 (1.5%)
T stage ^a^	
1	3 (6.5%)
2	12 (26.1%)
3	19 (41.3%)
4	12 (26.1%)
Nodal involvement ^a^	
No	16 (42.1%)
Nodes not sampled	7 (18.4%)
Yes	15 (39.5%)
M stage ^a^	
0	21 (58.3%)
1	15 (41.7%)

^a^ TNM staging was provided at the initial presentation.

**Table 2:** Adrenal and treatment characteristics.

	N = 66
Median size of largest adrenal metastasis, cm (IQR) ^a^	2.8 (2.1–4.5)
Median total size of adrenal metastasis, cm (IQR) ^a^	2.8 (2.1–4.9)
Median months to adrenal metastasis (IQR)	14 (5–37)
Metastatic adrenal laterality (overall)	
Bilateral	25 (37.9%)
Left	17 (25.8%)
Right	24 (36.4%)
Timing of bilateral adrenal involvement	
Metachronous	14 (21.2%)
Synchronous	11 (16.7%)
Unilateral	41 (62.1%)
Median months to contralateral adrenal metastasis (IQR)	6 (0–23)
FDG Avid	29 (43.9%)
Median SUV (IQR)	9.8 (5.7–15.5)
Adrenal biopsy	13 (19.7%)
Adrenalectomy	11 (16.7%)
Median months to adrenalectomy (IQR)	13 (0–19)
Status of contralateral adrenal gland at the time of SAbR	
Bilateral adrenal metastases	16 (24.2%)
Prior adrenalectomy	9 (13.6%)
Unaffected	41 (62.1%)
Median months to SAbR from initial cancer diagnosis (IQR)	30 (16–53)
Pre-SAbR systemic therapy	49 (74.2%)
Pre-SAbR IO therapy	19 (28.8%)
Adrenal SAbR laterality	
Bilateral	9 (13.6%)
Unilateral with unaffected contralateral adrenal gland	41 (62.1%)
Unilateral with bilateral metastases	7 (10.6%)
Solitary unilateral	9 (13.6%)
SAbR fractions, median dose/fraction	
1	1 (1.5%), 2000 Gy/fx
3	18 (27.3%), 1350 Gy/fx
4	1 (1.5%), 600 Gy/fx
5	44 (66.7%), 800 Gy/fx
10	2 (3.0%), 300 Gy/fx
SAbR intent ^b^	
Consolidative	15 (22.7%)
Curative	20 (30.3%)
Definitive	15 (22.7%)
Palliative	16 (24.2%)
SAbR approach	
IMRT	2 (3.0%)
SBRT	64 (97.0%)
Median BED-LQ, Gy (IQR)	128.2 (64.2–200.3)
Median BED-USC, Gy (IQR)	84.2 (63.0–160.1)

^a^ Size was calculated based on the largest diameter or the metastasis. Total size was calculated as a sum of the largest diameters of adrenal metastases. ^b^ The SAbR intent was defined as consolidative—radiation given in conjunction with or concurrent with systemic therapy in the setting of oligoprogression; curative—radiation given to monometastasis with the intent to cure the patient; definitive—in the setting of oligometastasis (1–5) and radiation given with the intent to provide maximum local control of the treated site; and palliative—radiation given for the purpose of palliation of sign or symptom.

**Table 3:** Logistic regression analysis for post-SAbR primary adrenal insufficiency.

9	N with PAI/Total	Odds Ratio (95% CI)	*p*
Age at diagnosis (per 5 years)	-	1.11 (0.75, 1.64)	0.60
Sex			
Female	2/18	reference	0.69
Male	7/47	1.40 (0.26, 7.47)	
Primary tumor site			
Lung	2/25	reference	0.28
Renal cell carcinoma	6/27	3.29 (0.60, 18.1)	
Other	1/13	0.96 (0.08, 11.7)	
M stage			
0	4/21	reference	0.94
1	3/15	1.06 (0.20, 5.64)	
Pre-SAbR systemic therapy			
Yes	5/48	0.38 (0.09, 1.62)	0.19
No	4/17	reference	
Pre-SAbR immunotherapy			
Yes	2/19	0.66 (0.12, 3.49)	0.62
No	7/46	reference	
Size of largest adrenal metastasis (per cm)	-	0.84 (0.54, 1.31)	0.45
Total size of adrenal metastasis (per cm)	-	1.01 (0.77, 1.34)	0.92
Timing of bilateral involvement			
Metachronous	4/14	7.80 (1.25, 48.8)	0.051
Synchronous	3/10	8.36 (1.18, 59.4)	
Unilateral	2/41	reference	
Adrenalectomy			
Yes	4/11	5.60 (1.21, 26.0)	0.028
No	5/54	reference	
Status of contralateral adrenal gland			
Bilateral adrenal metastases	4/15 ^¶^	14.6 (1.47, 144)	0.016
Prior adrenalectomy	4/9	32.0 (2.96, 346)	
Unaffected	1/41	reference	
Adrenal SAbR laterality			
Bilateral	4/9	8.16 (1.64, 40.6)	0.010
Unilateral	5/56	reference	
BED-LQ, Gy (per 10 Gy)	-	1.06 (0.97, 1.15)	0.19
BED-USC, Gy (per 10 Gy)	-	1.08 (0.96, 1.21)	0.19
Pre-SAbR cortisol (per μg/dL)	-	1.27 (0.93, 1.73)	0.13

One patient on chronic dexamethasone was excluded from this analysis. ^¶^ Of patients with bilateral adrenal metastases, only those treated with bilateral SAbR developed PAI and none of the patients with unilateral SAbR. Abbreviations used: PAI = primary adrenal insufficiency; SAbR = stereotactic ablative radiation.

**Table 4:** Characteristics of patients with post-SAbR primary adrenal insufficiency.

Age at Cancer Diagnosis/Sex	Primary Cancer	Adrenal Metastasis Side	Total Adrenal metastasis Burden (cm)	Adrenalectomy	SAbR	BED-LQ (Gy)	BED-USC (Gy)	Time from SAbR to PAI, Months	PAI CTCAE Grade	MR Deficiency
Pt 1: 60/M	Renal cell	Bilateral	5.9	No	Bilateral	200.3	173	12.5	4	No
Pt 2: 66/F	Renal cell	Right	1.5	No	Right	103.2	57.32	16.3	2	Yes
Pt 3: 75/M	Colorectal	Bilateral	5.5	No	Bilateral	231.8	192.23	4.3	2	Yes
Pt 4: 65/M	Lung	Bilateral	8.6	No	Bilateral	64.2	64.18	4.3	3	No
Pt 5: 45/M	Renal cell	Bilateral	2.2	Yes	Left	202.3	86.67	0.7	2	No
Pt 6: 59/M	Renal cell	Bilateral	3.1	Yes	Left	150.3	150.34	20.2	4	Yes
Pt 7: 69/F	Lung	Bilateral	2.4	No	Bilateral	64.9	59.64	2.8	2	No
Pt 8: 60/M	Renal cell	Bilateral	2.7	Yes	Right	246.1	78.42	6.3	1	No
Pt 9: 66/M	Renal cell	Right	2.5	Yes	Right	50.9	50.92	4.2	2	Yes

Abbreviations used: Pt = patient, F = female, M = male, SAbR = stereotactic ablative radiotherapy, BED = Biological Effective Dose; PAI = primary adrenal insufficiency, CTCAE = Common Terminology Criteria for Adverse Events, MR = mineralocorticoid.

**Table 5:** Survival after adrenal SAbR.

	6-Month Survival/ Failure (95% CI)	1-Year Survival/ Failure (95% CI)	Median Survival/ Failure (95% CI)
Cancer-specific survival	89.0% (78.3, 94.6)	74.8% (61.6, 84.0)	22.7 (17.7, 40.4)
Progression-free survival	82.9% (71.2, 90.2)	74.8% (61.5, 84.1)	80.0 (23.4-NR)
Adrenal insufficiency failure	8.4% (3.6, 19.1)	10.4% (4.8, 21.7)	Not reached

## Data Availability

Data contained were gathered under IRB protocol with institutional patient data; further inquiries can be directed to the corresponding author.
